# “Microbial metabolites in cardioprotection: an immune-engineered framework for heart failure and post-ischemic remodeling: a narrative review”

**DOI:** 10.1097/MS9.0000000000004529

**Published:** 2025-12-18

**Authors:** Asra Amjad, Asma Azam, Wadiha Shah, Umair Ali, Maryam Altaf, Muddassir Khalid, Muhammad Rizwan Javed

**Affiliations:** a Islamic International Medical College, Rawalpindi Pakistan (Riphah International University), Pakistan; b Department of Medicine, Karachi Medical and Dental College Karachi, Pakistan; c Department of Medicine, Gajju Khan Medical College, Swabi, Pakistan; d Department of Pharmacy, University of Swabi, Pakistan; e Department of Medicine, Karachi Metropolitan University, Pakistan; f Department of Medicine, Nishtar Medical University, Multan, Pakistan

**Keywords:** cardioprotection, gut-heart axis, heart failure, microbial metabolites, postbiotics, synthetic biology

## Abstract

**Background:**

The gut-heart axis is a new therapeutic target for cardiovascular diseases, which continue to be the leading cause of death worldwide. Metabolites originating from microorganisms have demonstrated significant impacts on inflammation, remodeling, and heart function.

**Objective:**

By assessing molecular pathways, therapeutic options, and translational barriers, this narrative review seeks to objectively examine the cardioprotective potential of gut microbial metabolites.

**Methods:**

We reviewed and summarized the most recent research on the impact of microbial metabolites, both beneficial and detrimental, on endothelium repair, fibrosis, immunometabolism, and cardiac bioenergetics. A systematic search was conducted using the phrases “gut-heart axis,” “microbial metabolites,” and “cardiovascular protection” in PubMed, Scopus, and Web of Science for the years 2018–2024. Preclinical and clinical research that met the eligibility requirements were vetted and narratively synthesized.

**Results:**

By modulating Nrf2, HDAC, and TGF-β/Smad, protective metabolites such as short-chain fatty acids, urolithins, and indoles have anti-inflammatory, anti-fibrotic, and mitochondrial-enhancing properties. On the other hand, oxidative stress and fibrosis are made worse by trimethylamine N-oxide and hydrogen sulfide. Postbiotic formulations, innovative delivery systems, and synthetic biology provide promising approaches for focused intervention. However, there are substantial translational obstacles due to pharmacokinetic, regulatory, and microbiota heterogeneity.

**Conclusion:**

Microbial metabolites produced in the gut are a promising and revolutionary class of cardioprotectants. Future cardiovascular treatments may be redefined by precision-targeted gut-heart axis modulation, although clinical validation and regulatory standardization are still crucial.

## Introduction

### Brief context of the gut–heart connection

The top killer in the world is cardiovascular disease (CVD), which accounts for roughly 31% of deaths worldwide^[1]^. There is increased evidence to suggest a fatal gut-heart axis, an area in addition to classical risk factors such as HT and hyperlipidemia, in which the intestinal microbiome and its products impact cardiac health through inflammation, immune, and neurohormonal pathways^[2,3]^. In heart failure (HF) or after myocardial infarction (MI), impairment of the gut barrier enables gut microbial endotoxin (microbial endotoxins such as lipopolysaccharide) entry into the bloodstream, culminating in systemic inflammation and the worsening of cardiac remodeling^[4,5]^. Observational research studies have reported changes in microbial diversity and composition (e.g., the lower abundance of *Faecalibacterium*) in patients with cardiovascular pathologies^[3,6,7]^.

**HIGHLIGHTS**

- The gut–heart axis is a novel therapeutic target in cardiovascular disease.
- Microbial metabolites modulate inflammation, fibrosis, and cardiac bioenergetics.
- Short-chain fatty acids, urolithins, and indoles show anti-inflammatory and cardioprotective roles.
- Trimethylamine N-oxide and hydrogen sulfide aggravate oxidative stress and fibrosis in the failing heart.
- Postbiotics, smart delivery, and synthetic biology offer translational potential.

### Focus on microbiota-derived metabolites as active modulators of cardiovascular function

Instead of focusing on the composition of the microbiome, recent studies have put their focus on the direct regulation of cardiovascular physiology of microbial metabolites, in particular^[2]^. In relation to cardiovascular health, microbial metabolites can be either beneficial or harmful. Short-chain fatty acids (SCFAs) such as acetate, propionate, and butyrate, which reduce blood pressure and inflammation by activating FFAR2/3, urolithin A (UA) and urolithin B (UB), which improve mitophagy and activate Nrf2 signaling to protect cardiomyocytes, indole derivatives, which maintain endothelial integrity, and secondary bile acids, which control vascular tone via nuclear receptors, are examples of protective metabolites. In contrast, harmful metabolites such as trimethylamine N-oxide (TMAO), which is generated from choline and carnitine, encourage thrombosis, atherosclerosis, and unfavorable remodeling after MI, and too much hydrogen sulfide (H_2_S) causes mitochondrial dysfunction and myocardial inflammation^[8-14]^.

### Rationale for focusing on microbial metabolites with therapeutic potential, not generalized dysbiosis

An intervention targeting specific microbial metabolites has several benefits compared to treating wide dysbiosis:

#### Mechanistic specificity

SCFA prevents deacetylases on histones of endothelium^[8]^. UA and UB induce mitophagy in cardiomyocytes, accomplished by forming Nrf2 pathways of PGC-1α^[7,10]^. TMAO increases the TGF beta/Smad pathway and stimulates fibrosis^[13]^.

#### Postbiotic strategies

Southern purification of metabolites can be used to perform precise dose, higher bioavailability, stable, and scalable production that does not need live organisms^[15]^.

#### Engineered delivery via synthetic biology

The gut environment can be genetically engineered to produce protective cardiovascular metabolites by the interaction of genetically engineered microbial consortia^[16]^.

#### Precision medicine platform

Metabolite profiling can be used to stratify patient risk and therapy personalization in accordance with the question of personalized cardiovascular care^[14]^. These facts cumulatively argue for a change toward therapeutic microbiome interventions in favor of strategies that directly employ the microbial products (metabolites) as the core cardiovascular therapeutics.

“Gut-heart axis,” “microbial metabolites,” “cardioprotection,” and “heart failure” were the search terms used to extract literature from PubMed, Scopus, and Web of Science (2018–2024). English-language preclinical and clinical researches were included. Information about translational implications, cardiovascular effects, and molecular pathways was retrieved.

In this review, the discussion of the microbiota-derived metabolites, as opposed to the broad dysbiosis term, will be used to develop a mechanistic basis that can be used to exploit these compounds in the treatment of patients with ischemia and HF. Critical evaluation of protective and pathogenic metabolites and molecular targets has led us to a new paradigm on the use of microbial metabolites as pharmacological agents in cardiovascular management. Building on this therapeutic and mechanistic justification, it is crucial to classify the critical microbial metabolites, both advantageous and detrimental, involved in cardiovascular regulation to comprehend their translational significance. This review is especially relevant because gut-derived metabolic therapies are still not well known, despite cardiometabolic disorders continuing to be the world’s leading cause of death. This effort attempts to connect fundamental immunometabolic research with precise cardiovascular therapeutic design by combining translational and mechanistic insights. Thus, the goal of this review is to thoroughly assess the cardioprotective potential of microbial metabolites derived from the gut by combining therapeutic innovations, translational barriers, and mechanistic evidence within the framework of precision immunocardiology.

## Microbial metabolites

### Classification and mechanistic relevance in cardiology

Microbial metabolites have been divided into protective and harmful groups, and they have different sources, host interactions, and cardiovascular effects.

#### Protective metabolites

1. **SCFAs**: The level of SCFAs is dependent on the level of dietary fibers, and these are made by the fiber-fermenting bacteria, which include *Faecalibacterium* and *Roseburia*. In HF, reduced SCFA-producing taxa are observed to associate with the microbial ecology and the balance of metabolites^[17]^.
2. **UA and UB**: These derivatives of ellagitannin are influenced by host intestinal enzyme potential-some generate much, and others produce nothing – this is why some people have high cardioprotection and others do not^[18]^.
3. **Indole derivatives and secondary bile acids**: These metabolites activate endothelial and hepatocytic receptors (e.g., FXR and TGR5) and thus indirectly regulate autonomic control and cardiac output through metabolic and neurohumoral pathways^[19]^.

#### Detrimental metabolite

1. **TMAO**: An excess of such foods containing choline or carnitine produces TMAO and vascular dysfunction, which are minimized by diminished excretion in renal dysfunction. Persistent pro-thrombotic signaling via TMAO leads to persistent myocardial fibrosis and platelet dysfunction^[20]^.
2. **Excess H_2_S**: Disruption of the gut barrier and excessive growth of sulfate-reducing bacteria result in the accumulation of toxic levels of H_2_S and compromise mitochondrial respiration in addition to stimulating oxidative stress in cardiomyocytes. A comprehensive summary of key microbial-derived metabolites, their sources, mechanistic pathways, and cardiovascular effects is presented in Table 1, providing a comparative overview of protective and detrimental metabolites discussed in this section.

**Table 1:** Protective and detrimental microbial metabolites in cardiovascular disease

Metabolite	Source	Mechanism of action	Cardiovascular effect
Short-chain fatty acids	Fiber-fermenting bacteria (*Faecalibacterium, Roseburia*)	Activates GPCRs (FFAR2/3), inhibits histone deacetylases	Vasodilation, BP reduction, inhibits inflammation
Urolithin A	Microbial metabolism of ellagitannins	Enhances mitophagy (Nrf2 activation), improves mitochondrial integrity	Reduces infarct size, protects cardiomyocytes
Urolithin B	Microbial metabolism of ellagitannins	Activates p62/Keap1-Nrf2 signaling cascade	Provides myocardial protection in ischemic injury
Indole-3-propionic acid	Tryptophan metabolism by gut microbes	Preserves endothelial function, inhibits inflammasome activation	Prevents cardiac inflammation
Secondary bile acids	Microbial transformation of bile acids	Activates FXR/TGR5 nuclear receptors	Modulates vascular tone and metabolism
Trimethylamine-N-oxide	Microbial metabolism of choline, carnitine	Activates TGF-β/Smad pathway, promotes platelet hyperreactivity	Atherosclerosis, fibrosis, platelet dysfunction
Hydrogen sulfide (excessive)	Overgrowth of sulfate-reducing bacteria	Damages mitochondrial bioenergetics, induces oxidative stress	Cardiomyocyte injury, worsens heart failure

#### Concentration-dependent effects of H_2_S

H_2_S has two physiological functions. It functions as a gaseous signaling molecule at physiological concentrations (<1 μM) that activates K_ATP channels and the Nrf2 pathway to increase mitochondrial efficiency, decrease oxidative stress, and induce vasodilation. On the other hand, too much H_2_S generated by sulfate-reducing bacteria or dysregulated enzymatic production (>10 μM) interferes with mitochondrial respiration by blocking cytochrome c oxidase, which causes contractile failure and cardiomyocyte death. H_2_S should therefore be viewed as a bimodal modulator, harmful at high concentrations but protective at low ones.

### Molecular pathways influencing cardiac structure and function

This part goes deeper into how the cellular and molecular processes underlying the responses to metabolites differ in the heart:

1. **Host receptor occupation**: SCFAs activate G-protein-coupled receptors (GPCRs; FFAR2/3 and GPR41) to normalize blood pressure and inflammatory tone, so bile acids occupy FXR/TGR5 to modulate metabolism of lipids and vasculature^[14]^.
2. **Epigenetic alterations**: Butyrate and propionate inhibit histone deacetylase (HDAC) in endothelial and cardiac cells, rewiring angiogenesis and antioxidant-associated gene expression.
3. **Fibrosis and inflammation**: TMAO augments TGF-beta/Smad pathways to lay down collagens and leads to NLRP3 inflammasome-mediated inflammation that aggravates remodeling of stiffened heart^[19]^.
4. **Mitochondrial control**: Urolithins trigger mitophagy and mitochondrial biogenesis by means of Nrf2-PGC1 alpha networks, enhancing cardiomyocyte health; meanwhile, excessive H_2_S leads to damaged mitochondrial membranes and ATP production.
5. **Systemic metabolic cross-talk**: The direction of metabolites is to regulate peripheral metabolism – liver, adipose, vascular signaling that affects cardiac substrate resources and systemic inflammatory milieu.

A larger theme of cellular reprogramming unites these molecular pathways, especially in immune control and cardiac bioenergetics, which are becoming more widely acknowledged as targets for treatments derived from microbes.

## Cellular and metabolic reprogramming by microbial metabolites

Small chemicals known as microbial metabolites, produced by gut bacteria, play a crucial role in changing host metabolic and cellular functions. These metabolites, such as bile acids, polyamines, tryptophan derivatives, and SCFAs, act as signaling molecules. They influence host tissue immune responses, energy metabolism, and gene expression^[21]^.

### Mitochondrial bioenergetics in ischemic myocardium (UA, butyrate, etc.)

Both in aged and in HF animals, UA, a derivative of the gut microbiota deriving from dietary ellagitannin metabolites, has been shown to markedly enhance mitochondrial quality and to provide cardioprotection. Oral UA treatment restored both systolic and diastolic heart function in preclinical mouse experiments by repairing the mitochondrial ultrastructure and activating the mitophagy pathways, including PINK1/Parkin and OPA1. Translating this to humans, a 4-month UA supplementation in healthy elderly individuals significantly reduced the circulating levels of ceramides – lipid markers that are associated with a higher risk of CVDs^[10]^. These results identify UA as a potential nutritional intervention that can be used to combat altered mitochondrial bioenergetics and metabolic derangement in cardiac aging and HF. It has been shown by research that a short-term administration of butyrate promoted ATP production and restored elevated ADP levels in the heart and mitochondrial oxidative phosphorylation in an animal model of metabolic heart disease. The effect of these changes was an enhanced heart contractile function, particularly during an increase in the workload. These results suggest that butyrate could restore cardiac energy balance and energetics by acting on mitochondrial bioenergetics, supporting the idea that the microbial metabolites could represent novel therapeutics for HF^[22]^.

### Immunometabolism modulation: SCFAs, indoles → macrophage, Treg, inflammasome effects

Butyrate is one of those critical SCFAs that kickstart the production of regulatory T cells in the colon. A study has shown that our gut microbiota actively communicates with the immune system, helping to keep everything in balance while also aiding in digestion. If this communication breaks down, it could lead to issues like heart disease, autoimmune disorders, and inflammation^[23]^. Malany and colleagues’ research from 2024 delves into how macrophages function in relation to the aryl hydrocarbon receptor (AHR), which gets activated by microbial metabolites like indole derivatives. Depending on the context, these gut-derived compounds, particularly those that come from breaking down tryptophan, can influence macrophages to adopt either pro-inflammatory or anti-inflammatory roles. The AHR’s ability to regulate immune responses highlights its crucial role in maintaining immune balance and its potential as a therapeutic target for conditions such as HF and CVDs that involve chronic inflammation^[24]^.

### *Relevance in myocardial recovery post-MI and chronic* HF

Yu and colleagues dive into the fascinating connection between the gut, immune system, and heart, particularly how this relationship impacts HF and the changes that occur in the heart after a heart attack. They highlight how immune responses that help heal heart tissue are influenced by the bacteria in our gut and the substances they produce. When there’s an imbalance in gut bacteria, a condition known as dysbiosis, it can lead to increased inflammation, which in turn can hinder proper heart remodeling and worsen HF. The researchers suggest that one promising way to boost heart repair and prevent HF from getting worse is by modifying gut microbiota or focusing on the metabolites produced by these microbes^[25]^. Myocardial repair is facilitated by metabolic reprogramming, but the next crucial frontier in microbiota-targeted therapies is tackling structural remodeling, especially cardiac fibrosis.

## Anti-fibrotic pathways: gut metabolites as modulators of cardiac remodeling

One of the main characteristics of unfavorable myocardial remodeling in HF is cardiac fibrosis, which causes ventricular rigidity and reduced contractility. New research indicates that metabolites originating from the gut microbiota can have indirect as well as direct anti-fibrotic effects, providing new therapeutic options to slow or reverse maladaptive remodeling in the context of post-ischemic and chronic HF.

### Evidence for microbial suppression of TGF-β/Smad and galectin-3 signaling in cardiac fibrosis

According to the study, spermidine supplementation in HF mice reduces unfavorable remodeling and stops additional elevation of galectin-3, but limiting spermidine significantly raises galectin-3 expression and impairs recovery. The study provides grounds that microbial-derived spermidine promotes cardiac protection via the gut-heart axis by suppressing galectin-3-mediated profibrotic signaling^[26]^. The TGF-β/Smad signaling pathway, a key driver of fibrotic remodeling, is modulated by gut microbiota, which has a substantial impact on myocardial fibrosis. SCFAs, which are beneficial microbial metabolites, reduce fibroblast activation, collagen production, and extracellular matrix deposition by suppressing TGF-β1 expression and blocking downstream Smad2/3 phosphorylation. On the other hand, dysbiosis and increased concentrations of pro-fibrotic metabolites such as TMAO boost TGF-β signaling, which in turn encourages fibrosis and fibroblast proliferation. As a result, the gut microbiota is a key regulator that can either suppress or exacerbate TGF-β-mediated myocardial fibrosis, making the gut-heart axis a viable target for treatment^[27]^. Inhibiting galectin-3 following MI lowers collagen accumulation and decreases inflammation. The TGF-β/Smad pathway is suppressed by this therapy, which lowers myofibroblast production and fibroblast activation. This results in improved ejection fraction and decreased ventricular dilatation, which enhances heart function and highlights galectin-3 inhibition as a potential tactic to prevent fibrosis and unfavorable remodeling^[28]^.

### Mechanistic targets in HFpEF and post-infarction ventricular remodeling

Pathological processes involving inflammation, fibrosis, metabolic dysfunction, and endothelial damage are shared by HF with preserved ejection fraction (HFpEF) and post-infarction ventricular remodeling. Key to these processes is the stimulation of profibrotic signaling cascades, especially the TGF-β/Smad pathway, which promotes overproduction of extracellular matrix components^[29]^. Inhibitors of galectin-3, a crucial TGF-β signaling modulator, have demonstrated potential in lowering fibrosis and enhancing ventricular function after myocardial damage^[28]^. The importance of the gut-heart axis as a mechanistic component to both HFpEF and post-infarction ventricular remodeling is highlighted by new data. Reduced SCFA-producing bacteria in HFpEF cause dysbiosis, which lowers SCFA levels and exacerbates inflammation, endothelial dysfunction, and fibrotic signaling through TGF-β/Smad pathways^[30]^. Microbial metabolites have important roles in maintaining endothelium integrity and vascular responsiveness, which are essential for preventing atherosclerosis and enhancing microvascular outcomes in addition to their effects at the cardiomyocyte level.

## Endothelial repair and microvascular integrity via microbial metabolites

### Enhancement of nitric oxide bioavailability, endothelial regeneration, and vascular compliance by butyrate and polyphenol-derived metabolites

The endothelium plays a crucial role in maintaining vascular health, regulating tone, maintaining barrier function, and controlling inflammation. Disruption of endothelial cells can lead to significant cardiovascular issues, including inflammation and vascular leakage. Recent research has highlighted the therapeutic potential of microbial-derived metabolites, particularly SCFAs, such as butyrate, in enhancing endothelial function and promoting vascular hemostasis^[31]^.

Butyrate, an SCFA produced by gut microbiota from dietary fibers, plays a significant role in promoting endothelial nitric oxide synthase activation through HDAC inhibition and AMP-activated protein kinase signaling. Additionally, microbial metabolites from dietary polyphenols, such as urothilins and phenyl-γ-valerolactones, contribute to vascular health by reducing oxidative stress, inflammation, and enhancing nitric oxide-mediated vasodilation^[31]^.

Together, these compounds support endothelial regeneration and improve vascular compliance, which are crucial for mitigating ischemic damage and attenuating the progression of HF. This emerging paradigm suggests that modulation of the gut microbiota and its metabolites may serve as a novel adjunctive strategy in post-ischemic cardiac care and chronic HF management^[32]^.

### Relevance in microvascular angina and early-stage atherosclerosis

The gut-heart axis has emerged as a pivotal area in cardiovascular research, highlighting the influence of gut microbiota and their metabolites on cardiovascular health. Microbial-derived metabolites, such as TMAO and SCFAs, play significant roles in modulating endothelial function, inflammation, and oxidative stress, which are critical in the development of conditions like microvascular angina and atherosclerosis. The dysbiosis, characterized by elevated TMAO and reduced SCFAs, contributes to endothelial dysfunction and inflammation, suggesting that restoring gut microbial balance through dietary changes, prebiotics, or probiotics could enhance vascular health and serve as adjunct therapies in CVDs. This perspective supports a paradigm shift in cardiovascular treatment strategies, emphasizing microbiota-targeted therapies for long-term vascular protection^[33]^. These metabolites are candidates for more comprehensive cardiovascular risk regulation because of their vascular and anti-inflammatory properties, as well as their significant role in forming cardiometabolic phenotypes such as insulin resistance, dyslipidemia, and hypertension.

## Microbial immunometabolites and the management of cardiovascular risk phenotypes

SCFAs, TMAO, and secondary bile acids are examples of metabolites generated from microorganisms that have a significant impact on cardiovascular control through their effects on inflammation, endothelial health, and myocardial remodeling. Clinical outcomes and the course of the disease are influenced by these metabolites, which are increasingly associated with hypertensive, dyslipidemic, and diabetic phenotypes. Probiotics, microbiota transplants, and dietary changes are examples of therapeutic strategies that target the gut-heart axis and have encouraging opportunities for risk reduction. Cardiometabolic care is reframed as a multi-organ, immunometabolic process from this gut-centric viewpoint^[34–38]^. Innovations in formulation, distribution, and regulation, areas where postbiotics, engineered consortia, and nanotechnology are starting to redefine therapeutic possibilities, are necessary to translate these biological effects into workable medicines.

## Emerging therapeutic platforms: beyond probiotics

### Postbiotic formulations: direct use of purified microbial

#### Metabolites

Purified microbial metabolites, such as butyrate and UA, have cardioprotective properties, but they do not fit the International Scientific Association for Probiotics and Prebiotics (ISAPP) requirement for postbiotics, which calls for inactivated microbial cells and/or their structural elements. As a result, this section makes a distinction between^[1]^ pure microbial metabolites, which are bioactive small molecules, and^[2]^ real postbiotics, which are complex preparations that contain or comprise components of non-viable bacteria that alter metabolic and immunological responses.

According to the ISAPP consensus, a postbiotic is defined as “a preparation of inanimate microorganisms and/or their components that confers a health benefit on the host.” In other words, postbiotics must contain non-viable microbial cells or cell components, but purified microbial metabolites alone do not qualify under this definition^[39]^. Unlike isolated microbial metabolites, actual postbiotic preparations must include inactivated microbial cells or cell components such as peptidoglycans, teichoic acids, lipoteichoic acids, or exopolysaccharides along with optional fermentation products like SCFAs or bioactive peptides^[40,41]^. These components act on the host by modulating immune responses, reinforcing gut epithelial integrity, and competitively excluding pathogens^[42,43]^. For instance, SCFAs like acetate, propionate, and butyrate promote anti-inflammatory effects by interacting with GPCRs and HDAC inhibition^[44]^. Other postbiotic elements, such as microbial-derived vitamins, antimicrobial peptides, and bacteriocins, contribute to systemic benefits beyond the gut, including modulation of metabolic and inflammatory pathways^[45]^. These diverse bioactive mechanisms make postbiotics a safe, stable, and emerging therapeutic strategy, especially in populations where live probiotic use may pose risks^[39,42]^. Although their cardiovascular applications are still under exploration, the biological plausibility built on gut-heart axis pathways supports further research in this field. A comparative overview distinguishing probiotics, postbiotics, and engineered microbial consortia, focusing on their definitions, advantages, and challenges in cardiovascular applications, is summarized in Table 2.

**Table 2:** A comparative overview distinguishing probiotics, postbiotics, and engineered microbial consortia, focusing on their definitions, advantages, and challenges in cardiovascular applications

Strategy	Definition (as per your text)	Advantages	Challenges/limits
Purified microbial metabolites	Pure forms of bioactive small molecules (such as SCFAs, urolithins, indoles, and secondary bile acids) generated by gut microorganisms that are devoid of biological components	Mechanistic specificity, defined dosing, scalable production, avoids live-cell risks	Short systemic half-life, poor bioavailability, may not reproduce full microbiota interactions
Probiotics	Live beneficial microorganisms	Gut colonization, broad-spectrum effects	Risk in immunocompromised individuals; variable colonization outcomes
Postbiotics	Inactivated microbial cells/components + optional fermentation products like SCFAs, peptides	Stable, safe in vulnerable populations, modulates immunity and barrier function	Regulatory ambiguity; requires standardization; not classified as “pure metabolites”
Engineered microbial consortia	Genetically modified bacteria designed to deliver therapeutic metabolites (ACE2, cholesterol-lowering enzymes)	Precision delivery of beneficial metabolites, targeted modulation	Complex design, scalability issues, long-term safety still under investigation

SCFA, short-chain fatty acid.

### Engineered microbial consortia and synthetic biology approaches

Cutting-edge synthetic biology is enabling the design of tailored microbial consortia and engineered probiotics to deliver precise therapeutic benefits, especially within cardiovascular medicine. Recent reviews describe the creation of genetically modified commensal bacteria capable of producing beneficial metabolites such as SCFAs and regulatory peptides *in situ*, aiming to modulate host metabolism and immune responses in hypertension and metabolic dysfunction^[46]^. In one notable preclinical study, researchers engineered *Lactobacillus paracasei* to express and deliver human Angiotensin-Converting Enzyme 2 (ACE2) in the gut, which led to a sex-specific reduction in blood pressure in female ACE2 knockout rats by lowering colonic angiotensin II levels, supporting the concept of gut-mediated modulation of systemic blood pressure regulation^[47]^. Another study describes an engineered synthetic consortium using *Escherichia coli* Nissle 1917 strains modified to express enzymes such as Isoflavone Synthase A (IsmA), Bile Salt Hydrolase (BSH), and Butyryl-CoA: Acetate CoA-Transferase (BCoAT), leading to a 43.6% reduction in serum cholesterol in mice fed a high-fat diet. Importantly, this consortium was equipped with an oleic acid-inducible system, ensuring that therapeutic gene expression occurred specifically under high-fat conditions. This targeted design also resulted in reduced inflammatory cytokines, improved gut barrier integrity, and optimized lipid metabolism, indicating its therapeutic relevance for cardiovascular risk modulation^[48]^. The development of synthetic gene circuits as programmable frameworks for precise medication delivery is highlighted in recent reviews in synthetic biology. In order to achieve the geographical and temporal specificity that traditional pharmacology is unable to achieve, these circuits incorporate modular sensor and regulatory components that may detect host or environmental signals and initiate the regulated release of therapeutic chemicals. In the cardiovascular domain, these circuits have been integrated into *Lactobacillus* and *E. coli* strains that have been designed to express enzymes like ACE2 or create cardioprotective metabolites (such SCFAs and antioxidant peptides) in response to local metabolic stimuli. Originally created for targeted cancer therapy, these systems represent the application of precision oncology concepts to immunocardiology, where gene-circuit-based microbial therapeutics could minimize systemic exposure while delivering metabolites directly within disease microenvironments^[49]^. Beyond specific examples, comprehensive reviews emphasize that engineered microbes can be programmed with synthetic gene circuits, biosensors, and regulated promoters to respond to *in vivo* signals and secrete therapeutic metabolites or proteins in a controlled manner^[49–51]^. Together, these bold approaches delivering ACE2 or cholesterol-lowering enzymes via engineered bacteria, and using inducible consortia tailored to metabolic states, illustrate a new therapeutic paradigm.

Scaling engineered microbial consortia presents significant challenges, including maintaining strain stability, genetic confinement, and consistent metabolite output in diverse gut conditions, despite preclinical promise. Similar to biologics, the production of such live therapeutics necessitates biocontainment procedures, scalable fermentation, and regulatory monitoring. In the absence of uniform safety and quality standards, practical implementation is still limited.

### Smart delivery systems for targeted release in cardiovascular patients

Delivering gut-derived microbial metabolites effectively to the heart and vasculature presents a significant challenge, especially when trying to avoid undesirable systemic effects and ensure sufficient concentrations at the target site. To address this, researchers have developed innovative delivery systems that enable precise, controlled release within cardiovascular tissues, particularly in conditions like ischemia, HF, or vascular inflammation.

One compelling approach involves biodegradable polymer and lipid nanoparticles, such as PLGA (poly-lactic-co-glycolic acid), liposomes, micelles, and dendrimers. These nanoparticles can be formulated to carry therapeutic molecules and have been studied extensively in models of myocardial injury. For example, in mouse models of ischemia–reperfusion, PLGA nanoparticles carrying cardioprotective agents like irbesartan or pioglitazone were preferentially taken up by inflammatory monocytes and delivered directly to injured myocardium, thereby reducing infarct size and improving post-infarction remodeling^[52]^. These same nanoparticle platforms are now being adapted to carry microbial metabolites such as butyrate derivatives or urolithins, aiming to leverage their anti-inflammatory and energy-protective effects in cardiac tissues. Liposomes sized around 70 nm showed even greater accumulation in ischemic heart tissue and localized to mitochondria, leading to improved heart function and reduced reperfusion injury, a phenomenon dependent on nanoparticle size. Liposomes, composed of phospholipid bilayers, are especially effective for microvascular targeting. Their ability to encapsulate both hydrophilic and hydrophobic molecules makes them ideal for slowly releasing growth factors, siRNAs, or signaling lipids that support microvascular regeneration and angiogenesis in ischemic heart disease^[53]^.

Another strategy takes advantage of the enhanced permeability and retention effect, where nanoparticles accumulate passively in inflamed or ischemic tissues. Lipid-based carriers and micelles, sometimes PEG-coated to extend circulation time, have shown improved accumulation within infarcted myocardium and atherosclerotic plaques^[54]^. In addition, nanoparticles conjugated with ligands such as antibodies against the Angiotensin II Type 1 receptor, or hyaluronan to target inflammatory macrophages, have achieved active targeting, delivering their payload directly to diseased cardiac tissue, minimizing off-target exposure, and suggesting potential for directing SCFA or microbial metabolite payloads to the cardiac immune microenvironment. Emerging research focuses on stimuli-responsive carriers, which release therapeutic agents in response to physiological cues such as shear stress, pH changes, or ultrasound activation. For instance, shear-activated nanoparticles are engineered to open and release their contents in regions of disturbed blood flow, like stenotic arteries or aneurysms. Ultrasound-assisted delivery also enhances deposition of nanoparticles such as mesoporous silica in targeted tissues for controlled release^[55]^.

In parallel, oral butyrate supplementation, a naturally occurring microbial metabolite, was shown to protect against myocardial ischemia–reperfusion injury in rats via a gut–brain neural circuit, reducing oxidative stress, apoptosis, and inflammation through vagal modulation^[56]^. Meanwhile, in a large animal (porcine) model, intravenous butyrate infusion significantly increased cardiac output and induced vasorelaxation in coronary arteries, demonstrating its direct hemodynamic benefits^[57]^.

Together, these elegant platforms, nanoparticles engineered for passive and active targeting, liposomal vehicles for microvascular delivery, responsive designs triggered by local cues, and natural metabolite supplementation, provide a robust toolkit for delivering microbial metabolite therapies to cardiovascular tissues. By enhancing bioavailability, optimizing localization, and safeguarding systemic safety, these technologies hold great promise for translating gut-derived metabolic treatments into effective interventions for MI, HF, and vascular disease.

### Regulatory and clinical trial considerations

Despite growing interest in postbiotic therapies for CVD, no unified regulatory framework currently exists for these agents. The ISAPP defines postbiotics, although regulatory bodies such as the FDA and EMA have not yet adopted clear definitions or approval pathways for postbiotics, making it difficult to classify them consistently as drugs, biologics, or supplements^[39,58]^. A critical regulatory challenge lies in the need for precise characterization of identity, purity, and mechanism of action of postbiotic compositions. Since they consist of non-viable microbial components and possibly metabolites, consistency in production is vital but currently lacking, with no established quality control or manufacturing standards across the industry^[39,59]^. Pharmacokinetic and pharmacodynamic evaluation presents another complexity: individual variation in gut microbiota leads to heterogeneous postbiotic absorption and response, complicating dose standardization. To date, there are limited biomarkers to track systemic or tissue-specific bioavailability in clinical settings^[59,60]^.

Oral butyrate, for instance, has a limited systemic availability due to its fast intestine degradation. To improve systemic bioavailability and distal gastrointestinal distribution, formulation improvements, such as lipid-nanoparticle carriers, butyrate prodrugs, and enteric-coated capsules, are being tried. For polyphenol-derived metabolites like UA, comparable micellar or encapsulating methods may circumvent first-pass metabolism and promote targeted cardiac absorption.

Designing clinical trials for postbiotic therapies presents multiple challenges that must be addressed to ensure their successful translation into cardiovascular treatments. A major hurdle is the significant variability in individual gut microbiomes, which affects the systemic absorption and bioactivity of microbial metabolites. Consequently, dosing protocols must be personalized or stratified to accommodate this interindividual microbiome diversity^[39,59,60]^.

Currently, only a few early-phase human clinical trials have begun evaluating cardiometabolic postbiotics, such as SCFAs or UA, in cardiovascular patients. None of these studies have progressed beyond Phase II to late-stage Phase III trials for conditions like HF or post-MI, highlighting the substantial translational gap that remains^[39,61]^.

To address these challenges, stakeholders have proposed several critical frameworks. First, there is a pressing need for standardized product definitions and nomenclature, as outlined by the ISAPP consensus guidelines, to ensure regulatory clarity and consistent scientific communication. Second, Good Manufacturing Practice-level protocols and reproducibility standards must be established to guarantee that postbiotic formulations contain reliable metabolite concentrations with batch-to-batch consistency. Finally, the development of adaptive clinical trial designs that stratify participants based on microbiome profiles, utilize validated mechanistic biomarkers, and implement precision dosing strategies is essential to overcoming the inherent variability in host–microbiota interactions and optimizing therapeutic outcomes^[39,59,60]^. In order to go from theory to practice, a number of translational bottlenecks still limit real-world application, despite conceptual and technological advancements.

## Clinical trials and translational gaps

Despite growing preclinical evidence, translational studies assessing microbial-derived metabolites in CVD remain limited. Most data are derived from early-phase animal models, with human clinical trials still in their infancy. One pivotal preclinical study by Gong *et al* demonstrated that oral butyrate supplementation provided robust protection against myocardial ischemia–reperfusion injury in rats. The study revealed that butyrate’s cardioprotective effects were mediated through a gut-brain neural circuit involving vagal nerve activation, which subsequently suppressed oxidative stress, apoptosis, and inflammatory cytokine production within the myocardium. Notably, this study highlighted the systemic neuromodulatory role of butyrate, suggesting a therapeutic avenue beyond direct metabolite delivery^[56]^.

Building on this, Li *et al* investigated intravenous administration of butyrate in a large animal (porcine) model of cardiac dysfunction. Unlike the oral route, IV butyrate directly modulated vascular tone, inducing vasorelaxation in coronary arteries and enhancing cardiac output. This study emphasized butyrate’s hemodynamic benefits, particularly its capacity to influence peripheral vascular resistance, thereby alleviating cardiac workload. Importantly, it showcased the feasibility of systemic butyrate infusion as a therapeutic intervention in acute cardiac settings^[57]^.

Notably, one clinical crossover trial investigated UA in a small group of HF patients with reduced ejection fraction (HFrEF). In this randomized, double-masked, placebo-controlled crossover design, 10 patients received 500 mg UA twice daily for 4 weeks, followed by a 2-week washout before switching to placebo (or vice versa). The study found no significant improvements in echocardiographic indices (such as LVEF and LVEDD), nor in biomarkers like pro-BNP or CRP. Only HDL-C showed a modest increase compared to placebo. The authors concluded that higher doses, longer duration, and larger cohorts are needed^[62]^.

Collectively, these studies, while promising, underscore the current translational gap. Most evidence remains confined to preclinical or early-phase human trials, lacking large-scale randomized controlled trials (RCTs) necessary for clinical approval. Table 3 consolidates key findings from preclinical and early-phase clinical trials that have evaluated microbial metabolites for cardiovascular interventions, highlighting both their therapeutic potential and current translational limitations. Moreover, variability in dosing regimens, bioavailability across different delivery routes, and lack of standardized biomarkers for efficacy assessment continue to hamper the broader clinical adoption of postbiotic therapies in cardiovascular medicine.

**Table 3:** Summary of preclinical and clinical trials evaluating microbial metabolites in cardiovascular models

Metabolite	Study model	Key outcome	Limitations
Butyrate (oral)	Rat ischemia–reperfusion model	Cardioprotection via gut-brain neural circuit: reduced oxidative stress, apoptosis, and inflammation	Preclinical data; needs human studies
Butyrate (IV infusion)	Large animal (porcine) model	Increased cardiac output; induced vasorelaxation in the coronary arteries	No human clinical trials yet
Urolithin A	Human crossover trial (*n* = 10)	No significant improvement in LVEF or biomarkers; slight HDL increase after 4 weeks of supplementation	Small sample size, short trial duration

Despite the lack of significant cardiac improvement in HFrEF patients, the UA crossover trial’s mild HDL-C rise and lack of side effects point to a dose-response potential. Similar small human trials of SCFAs in metabolic syndrome and hypertension showed decreased blood pressure and improved endothelial function (Haghikia et al., 2022; Amiri et al., 2022), suggesting that combination therapy and optimal dosing duration may be necessary for metabolite efficacy. Integrating these early-phase findings implies a favorable translational trajectory from metabolic control to cardiovascular benefit.

## Research gaps and future directions

The development of microbial-metabolite therapeutics depends on validated mechanistic biomarkers. Potential candidates include new neurocardiac indicators as neurofilament light chain (Geurts et al., 2023) reflecting cardio-neural interaction, galectin-3 and NT-proBNP as fibrosis-linked efficacy markers, and plasma TMAO and butyrate levels as exposure signals. By including these biomarkers into early-stage studies, the gut-heart axis paradigm’s precision-medicine goal would be strengthened by making pharmacodynamic evaluation and patient categorization easier.

Even with the encouraging preclinical and early translational evidence, there are still a number of significant research gaps. First, the stratification of individuals who are likely to respond to particular microbial metabolites is hampered by the absence of standardized metabolomic profiling, which restricts precision-targeted medication. Second, the requirement for tailored therapies based on microbiome phenotyping is highlighted by the significant barrier to therapeutic generalization posed by the variation in gut microbiota composition among individuals and populations. The development of nanoformulations and stimuli-responsive carriers that guarantee site-specific action is necessary since the bioavailability and tissue-specific distribution of microbial metabolites, especially to heart tissue, have not yet been optimized. Fourth, large-scale RCTs are desperately needed to establish long-term efficacy, safety, and dosing protocols. Clinical trials are still in their infancy, with the majority of studies concentrating on surrogate biomarkers or small, short-duration cohorts. Furthermore, licensing processes and manufacturing standards are complicated by regulatory ambiguity around postbiotic classification (as medicines, biologics, or nutraceuticals). Last, as synthetic biology develops, strong regulations and interdisciplinary supervision are needed to address biosafety, horizontal gene transfer, and ethical issues pertaining to constructed microbial consortia. To create a genuinely customized cardiometabolic care paradigm, future studies should also investigate integrative frameworks that include microbiota editing, AI-guided diagnostics, and multi-omics. The instability of microbial formulations, interindividual microbiome variability impacting metabolite bioavailability, and the lack of regulatory standards for postbiotic or engineered-microbe classification are important translational barriers. Clinical translation will need addressing issues through harmonized guidelines and stratified trial designs. Small sample sizes, brief follow-up periods, and variation in study designs significantly restrict the available information. To confirm clinical efficacy, future large-scale RCTs combining metabolomic and microbiota characterization are required.

## Conclusion

This review provides a shift from conventional paradigms in the treatment of HF and post-ischemic stroke by synthesizing new data on the function of metabolites produced from gut microbes as novel cardioprotective drugs. TGF-β/Smad pathway modulation, Nrf2 activation, HDAC inhibition, and GPCR signaling are the primary mechanisms by which protective metabolites like SCFAs, urolithins, indoles, and bile acid derivatives exhibit strong mechanistic effects, such as anti-inflammatory, antioxidant, antifibrotic, and mitochondrial-stabilizing qualities. On the other hand, harmful metabolites like H_2_S and TMAO intensify oxidative stress, cardiac remodeling, and fibrosis, highlighting the therapeutic significance of focused metabolic modification. Additionally, the review emphasizes the translational potential of postbiotic formulations, synthetic biology platforms, and intelligent delivery systems that improve patient specificity, cardiac targeting, and bioavailability. All of these results demonstrate the therapeutic potential of microbiota-based strategies in cardiovascular treatment. With the majority of the supporting evidence coming from animal research or early-stage human trials, this paradigm is still primarily experimental. Microbial metabolite-based approaches have the potential to go from being adjuncts to being the first line of treatment for ischemic heart disease and HF. In order to achieve this goal, a strong pipeline from clinical validation to mechanistic knowledge is essential. The complete integration of gut-derived medicines into cardiovascular therapy would require not only technological innovation and scientific rigor but also ethical vigilance and clear regulations.

TITAN Guidelines: This manuscript complies with TITAN Guidelines, 2025, declaring no use of AI^[63]^.

## Data Availability

This narrative review is based on previously published literature. No new data were generated or analyzed. Therefore, data sharing does not apply.
